# Supplementary material for: Thyroid Hormone Disruptors Interfere with Molecular Pathways of Eye Development and Function in Zebrafish
Source: Int J Mol Sci. 2019 Mar 27;20(7):1543. doi: 10.3390/ijms20071543 (PMC6479403; doi:10.3390/ijms20071543)
Supplement: Supplementary file 1 [file ijms-20-01543-s001.pdf]

Supporting information

Thyroid hormone disruptors interfere with molecular pathways of eye development and function in zebrafish

*Baumann, Lisa[1,2]; Segner, Helmut[2]; Ros, Albert[3]; Knapen, Dries[4]; Vergauwen, Lucia[4,5]

*corresponding author (lisa.baumann@uni-heidelberg.de)

1. Cluster analysis with subsequent analysis of enriched GO classes

The list of all differentially expressed transcripts was used to perform a cluster analysis in MEV (k-means – pearson correlation), resulting in 5 clusters. These 5 clusters were further analyzed in GOrilla. The transcript list from one cluster was set as target list and compared to a background list, consisting of all transcripts included in the 5 clusters to determine how the specific cluster is distinct from the remaining clusters in terms of affected GO classes.
Each cluster and its most enriched GO classes are listed in the following.

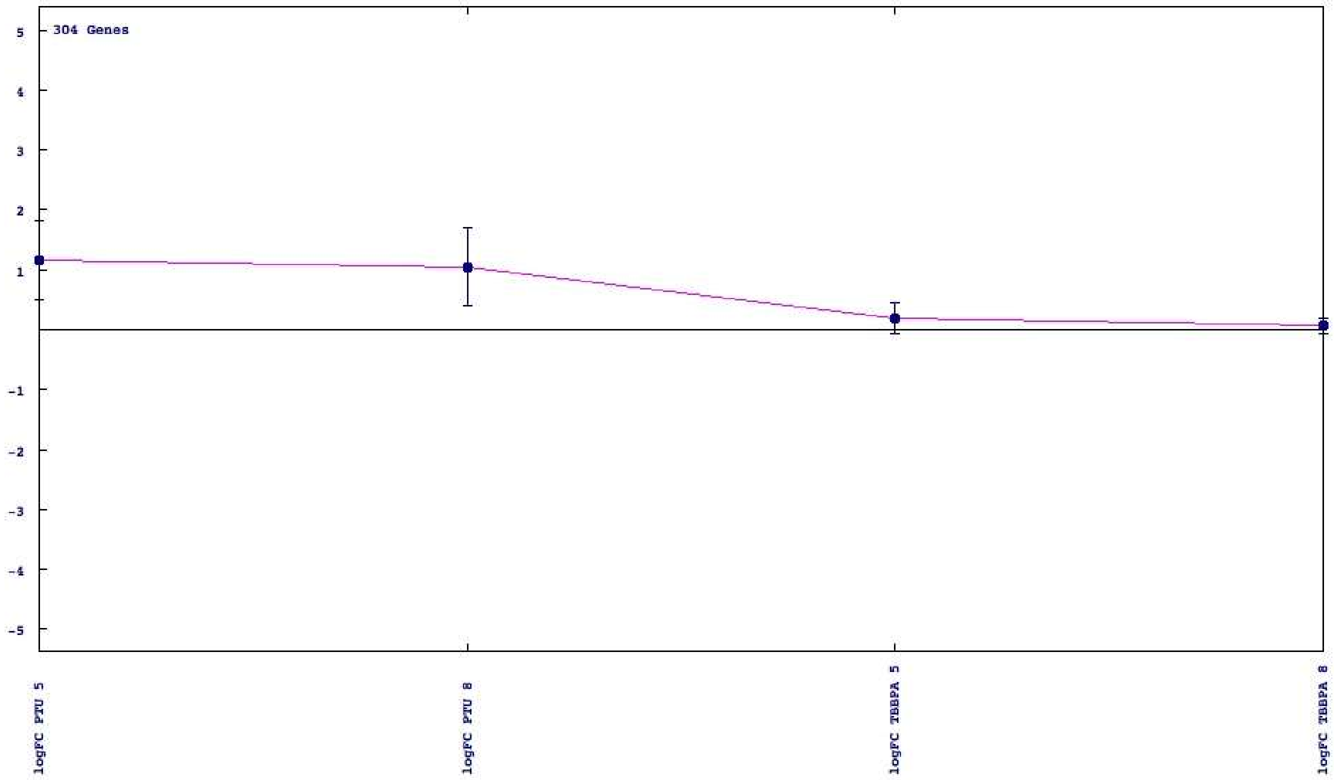

Fig. S1: cluster 1

(logFC = binary logarithm of the fold change)

Most enriched GOs for cluster 1 vs clusters 1-5:

proteolysis involved in cellular protein catabolic process
protein folding
tissue development
cell redox homeostasis
regeneration
positive regulation of cellular component organization
positive regulation of protein complex assembly

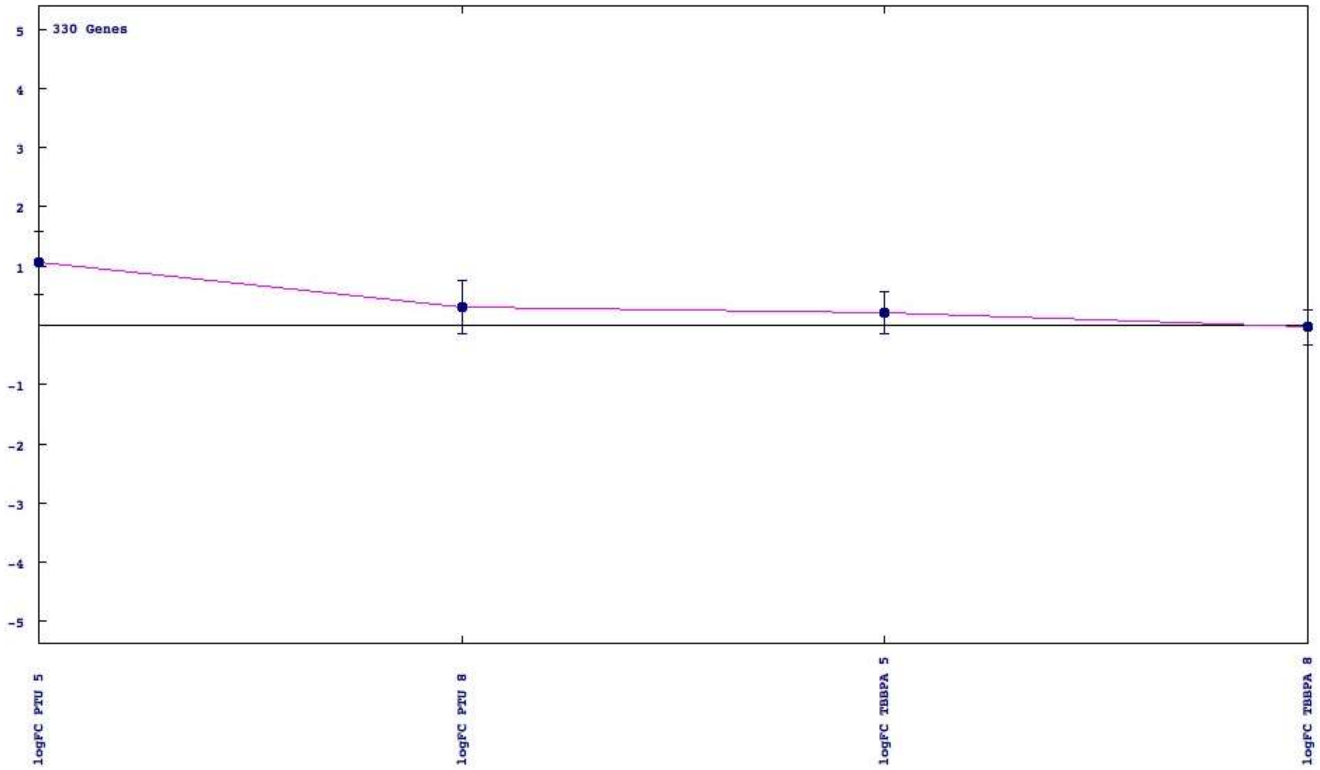

Fig. S2: cluster 2

(logFC = binary logarithm of the fold change)

Most enriched GOs for cluster 2 vs clusters 1-5:
RNA metabolic process
ncRNA metabolic process
nucleic acid metabolic process
tRNA metabolic process
cellular nitrogen compound metabolic process
regulation of metabolic process
regulation of macromolecule metabolic process
regulation of primary metabolic process

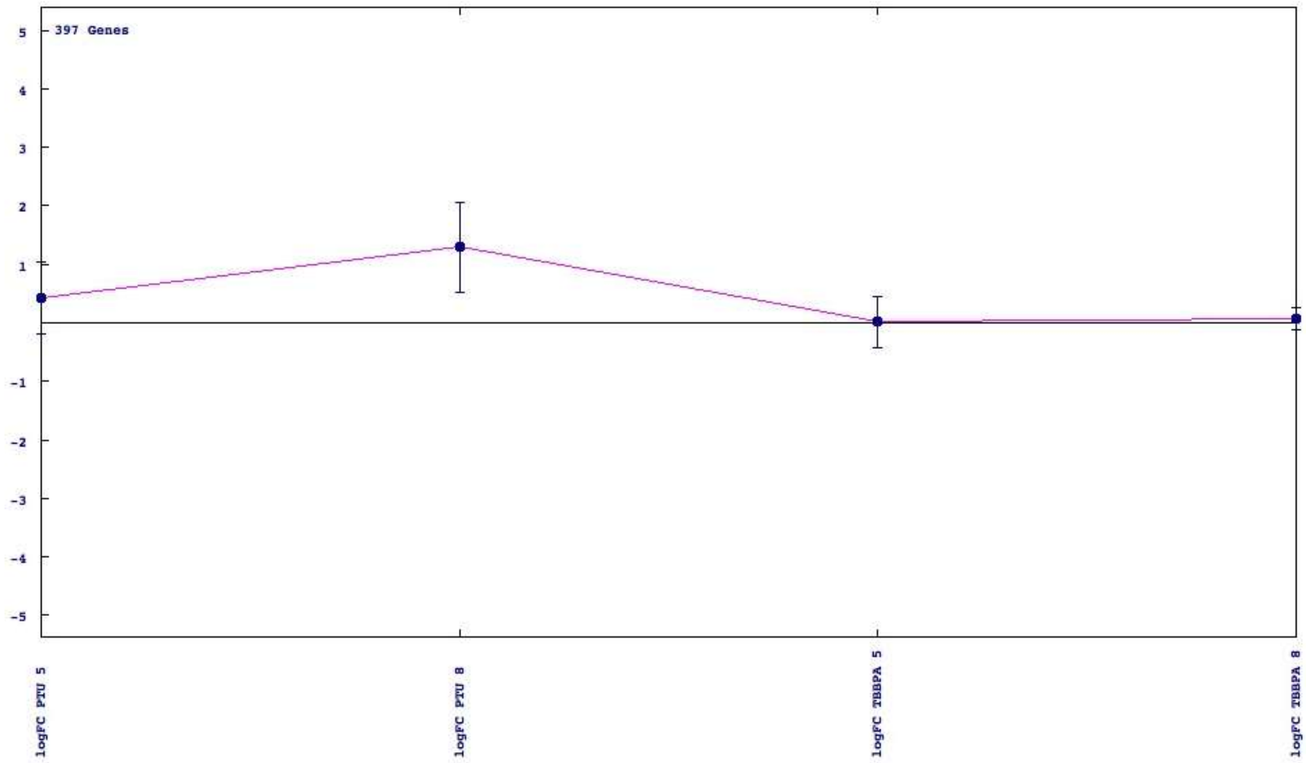

Fig. S3: cluster 3

(logFC = binary logarithm of the fold change)

Most enriched GOs for cluster 3 vs clusters 1-5:
proteolysis
immune system process

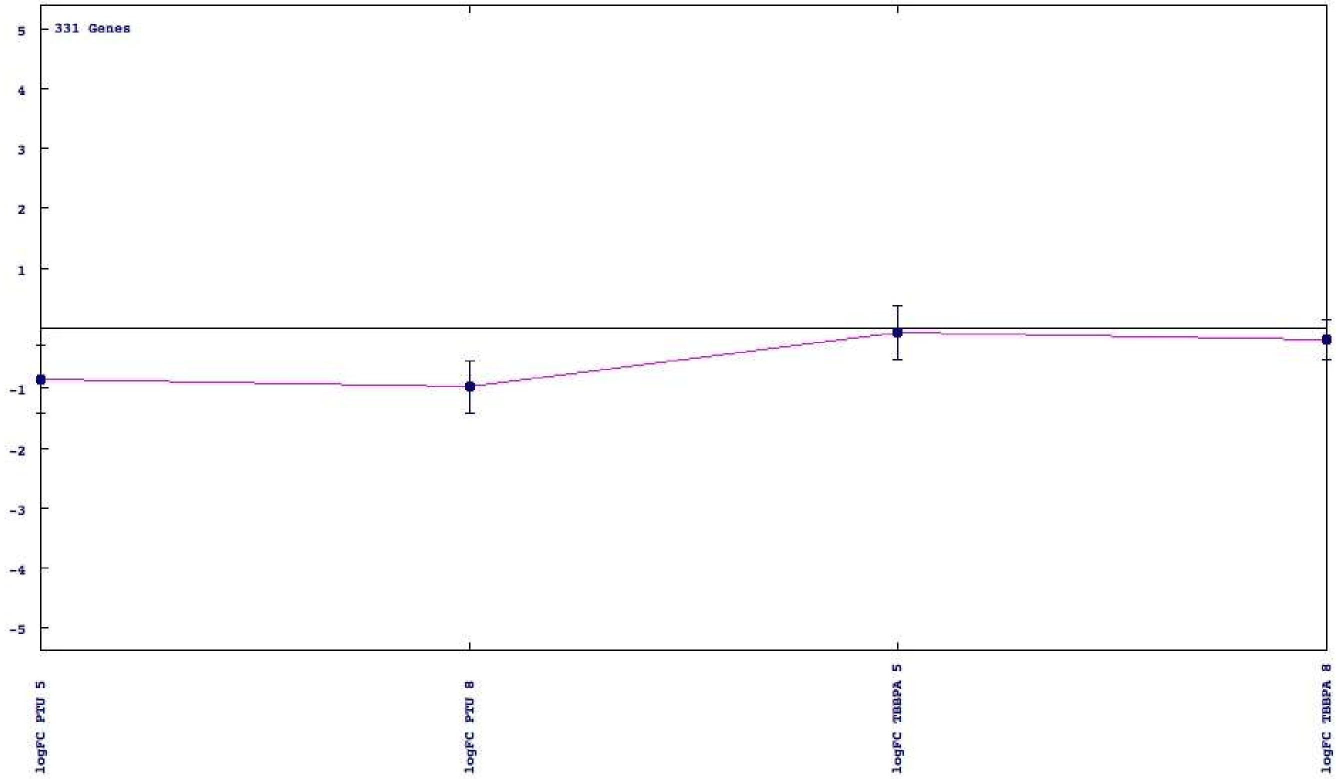

Fig. S4: cluster 4

(logFC = binary logarithm of the fold change)

Most enriched GOs for cluster 4 vs clusters 1-5:
hormone-mediated signaling pathway
steroid hormone mediated signaling pathway

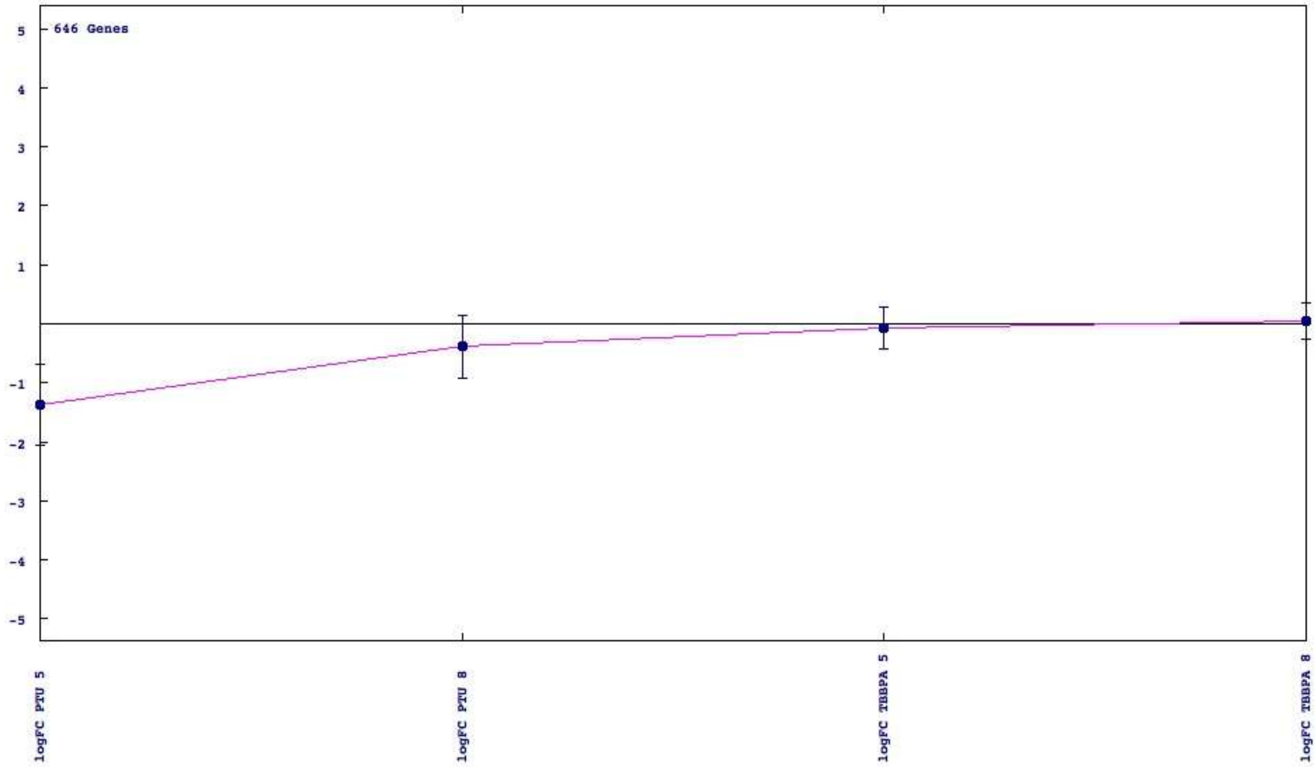

Fig. S5: cluster 5

(logFC = binary logarithm of the fold change)

Most enriched GOs for cluster 5 vs clusters 1-5:
sensory perception
neurological system process
sensory perception of light stimulus
visual perception
single-organism catabolic process

2. Heat maps of selected GO classes resulting from cluster analysis

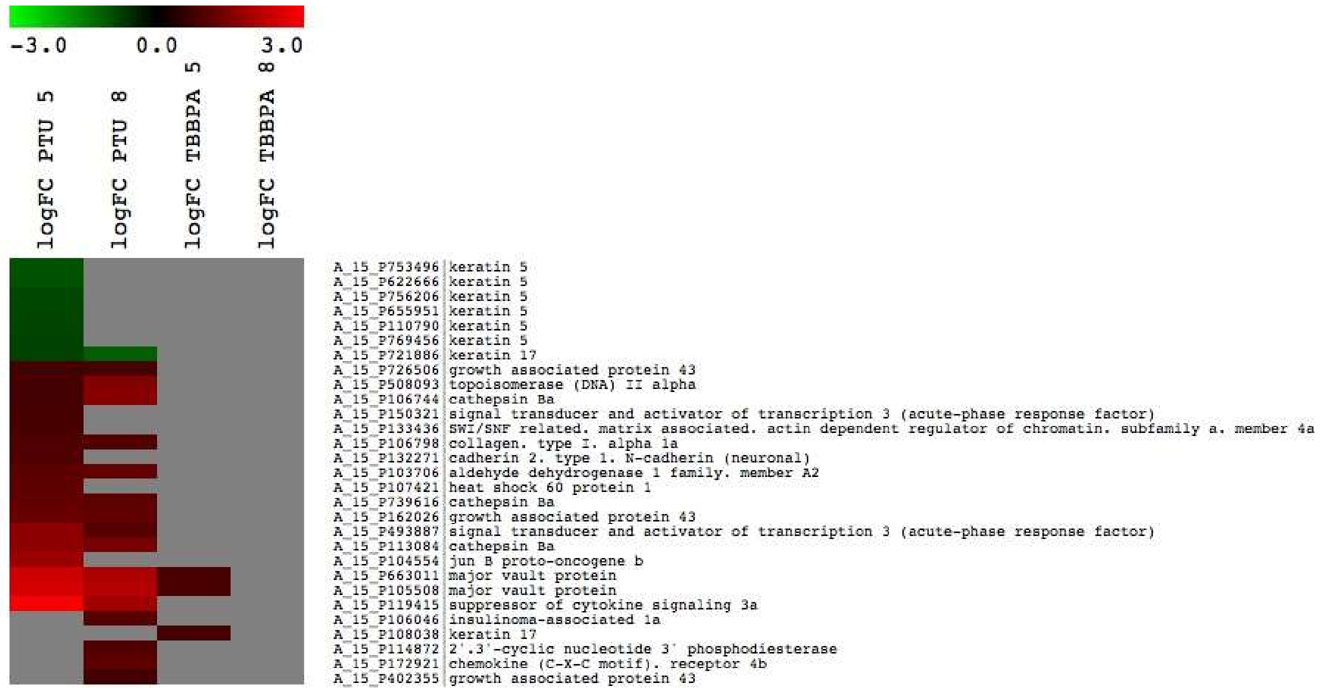

Fig. S6: GO class regeneration from cluster 1

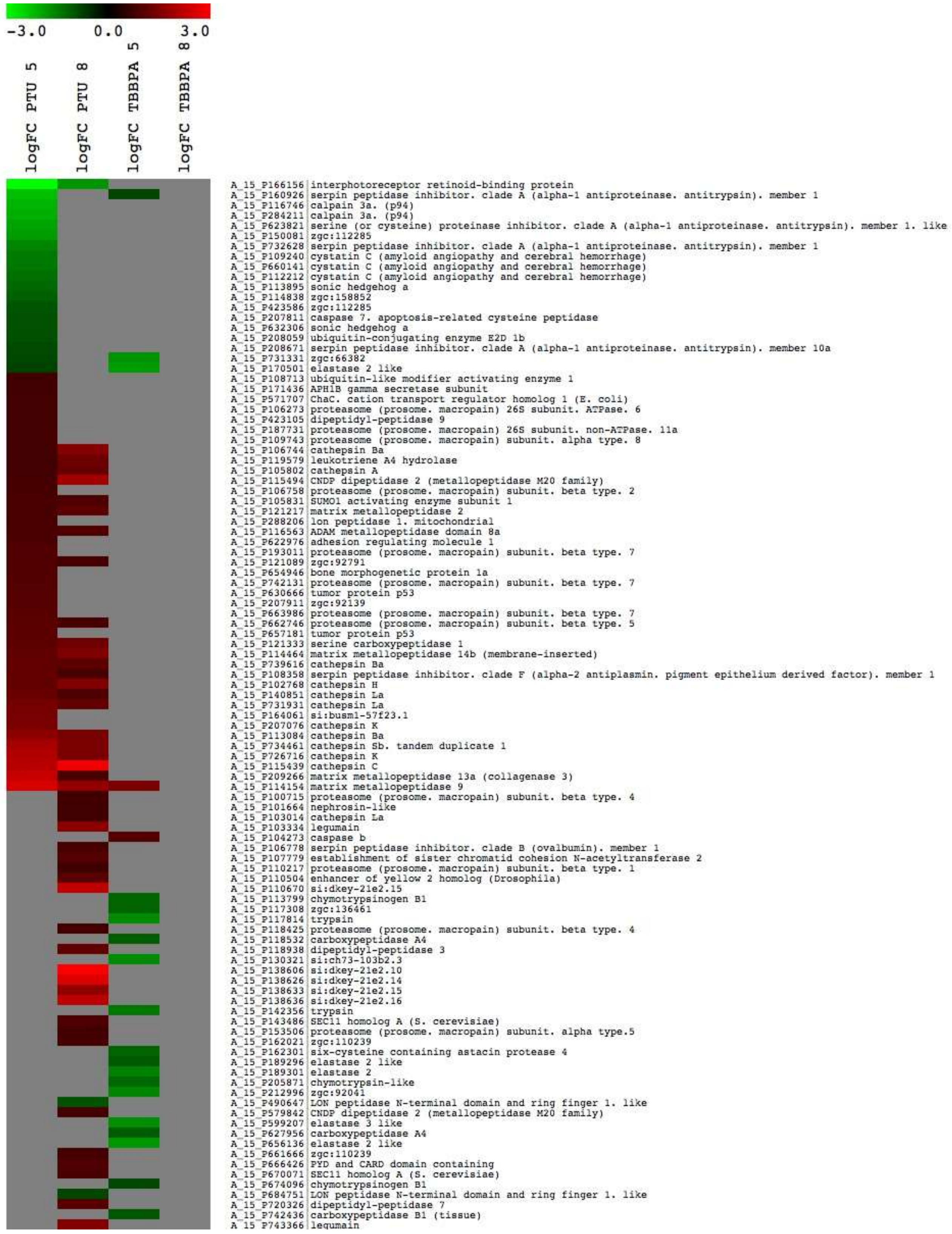

Fig. S7: GO class proteolysis from cluster 3

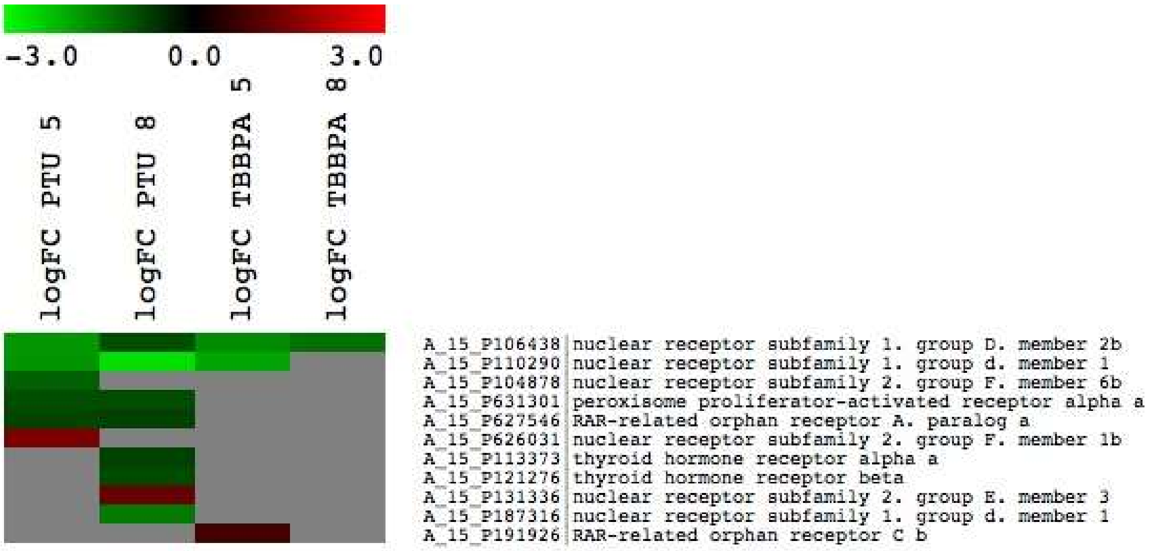

Fig. S8: GO class hormone-mediated signaling pathway from cluster 4

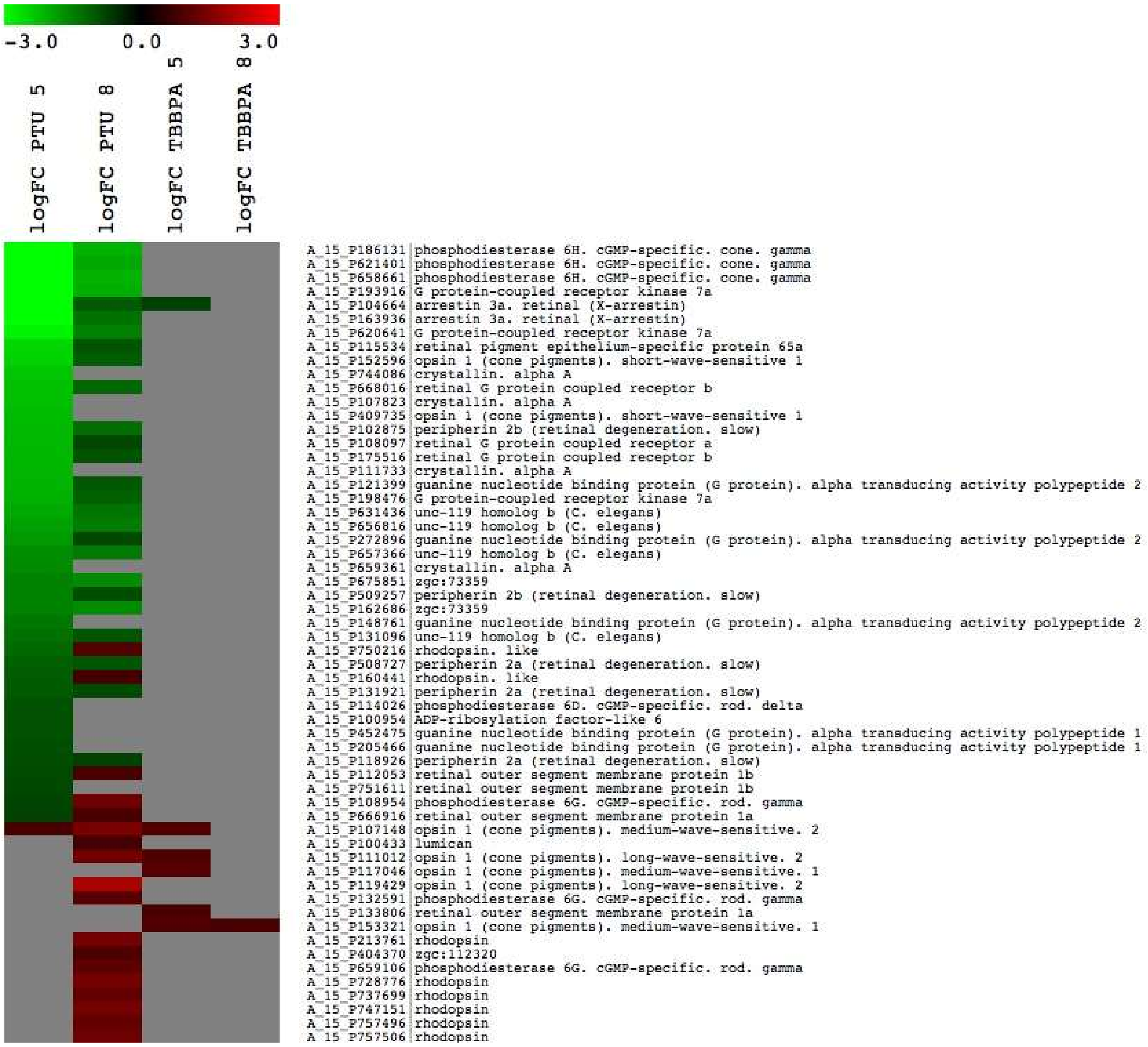

Fig. S9: GO class sensory perception from cluster 5

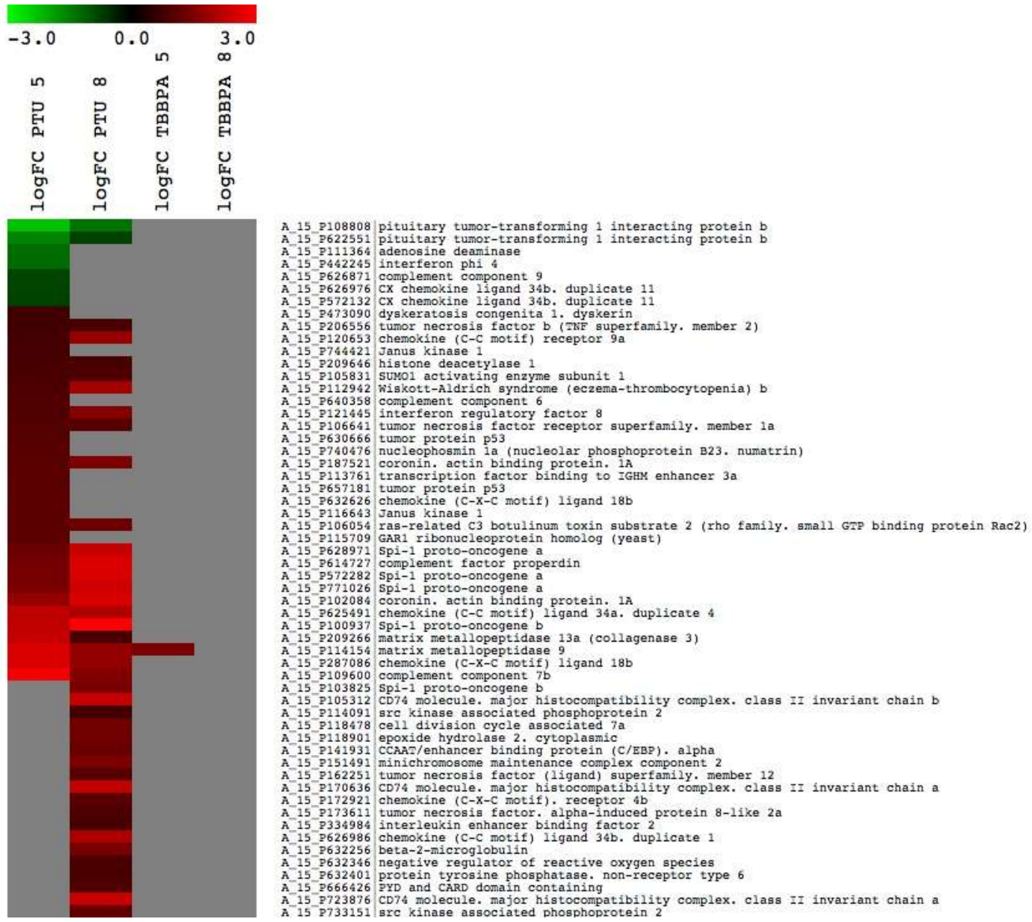

Fig. S10: GO class immune system process from cluster 3
